# Update on the role of pharmaceutical-grade chondroitin sulfate in the symptomatic management of knee osteoarthritis

**DOI:** 10.1007/s40520-019-01253-z

**Published:** 2019-06-26

**Authors:** Germain Honvo, Olivier Bruyère, Jean-Yves Reginster

**Affiliations:** 1 WHO Collaborating Centre for Public Health Aspects of Musculo-Skeletal Health and Ageing, Department of Public Health, Epidemiology and Health Economics, University of Liège, Quartier Hôpital, Avenue Hippocrate 13, CHU B23, 4000 Liège, Belgium; 2 Chair for Biomarkers of Chronic Disorders, Biochemistry Department, College of Science, King Saud University, Riyadh, 11451 Saudi Arabia

**Keywords:** Osteoarthritis, Chondroitin, Pain, Function, Safety, Treatment

## Abstract

Osteoarthritis (OA) is the most prevalent musculoskeletal disease and a major cause of negative relevant outcomes, associated with an ever-increasing societal burden. Pharmaceutical-grade chondroitin sulfate (CS) was repeatedly reported to reduce pain and improve function in patients with knee OA. This treatment was also shown to be cost-effective, compared to placebo, up to 24 months. However, controversies still persist regarding the usefulness of CS for patients with knee OA, mainly due to inconsistent reports from various clinical trials. In this literature review, we aimed to summarize the main most recent findings on the efficacy and safety of CS in OA. Based on the results of studies presenting a low risk of bias, the most recent meta-analysis shows that only the pharmaceutical-grade CS may be considered as an appropriate background treatment for the management of knee OA. Evidence from another recent meta-analysis, using data from full safety reports, confirms the good safety profile of CS in OA. This new evidence on efficacy and safety suggests that recommendations for the use of CS in patients with knee OA cannot be extrapolated to other low-grade preparations as generics, nutraceutical-grade or over-the-counter preparations.

## Introduction

Osteoarthritis (OA) is the most prevalent musculoskeletal disease and a major cause of negative, clinically relevant outcomes including, but not exhaustively, loss of functional capacity, pain and disability, which are all associated to an ever-increasing societal burden [1]. Several respected scientific societies published recommendations for the management of OA. Most of them focused on OA of the lower limbs [2–5] reflecting the medical and economic impact of knee and hip OA on the ageing population [6]. Albeit all evidence-based guidelines agree that the medical management of OA includes both non-pharmacological and pharmacological treatment modalities, differences are observed on the choice of drugs to be used and on the prioritization of the currently available medications [7]. Such discrepancies are common for the assessment of the efficacy of symptomatic slow-acting drugs for OA (SYSADOAs) in the management of knee OA [7, 8]. They mostly reflect heterogeneity of the experts panels involved or geographical differences in the availability of chemical entities [7, 8]. However, evidence of safety and efficacy seems to be provided for prescription-grade formulations of chondroitin sulfate (pCS) and crystalline glucosamine sulfate (pCGS) [5], which cannot be extrapolated to generics, nutraceutical-grade or over-the-counter (OTC) products [9–11].

The objective of this literature review is to summarize the main findings from the studies conducted to assess the effects of pCS on pain and function in knee OA, as well as recent findings on safety of CS in OA.

## Methods

In accordance with what was previously shown for GS [12], we recently published the outcomes of a comprehensive meta-analysis concluding that inconsistencies observed in the outcomes of randomized, placebo-controlled trials assessing the effects of CS in knee OA were explained by the risk of bias, brand and study size [13]. For the purpose of this literature review summarizing the best evidence on pCS in OA, we only considered the studies identified as presenting a low risk of bias in our recent systematic review and meta-analysis [13]. A summary of each of these studies was made, presenting their main characteristics and relevant efficacy results. To allow comparison between evidence on efficacy of pCS from different studies, the outcomes of these studies were also expressed as standardized mean differences (SMD) with 95% confidence interval (95% CI), as reported from our latest efficacy meta-analysis [13]. We also summarized the main findings from our latest systematic review and meta-analysis that reassessed the safety of CS in patients with OA [14].

## Results

### Symptomatic effects of pCS in knee OA

Our recent meta-analysis on the efficacy of CS [13] identified thirteen studies that assessed the effect of CS on pain in OA and which were ranked as presenting a low risk of bias. In total, 1694 patients received CS and 1717 patients were treated with a placebo. The overall effect size for pain (ES, fixed effect model) was − 0.18 (95% CI − 0.25, − 0.12; *I*^2^ = 71%). A subgroup analysis assessing the effect of CS according to manufacturer (i.e., the brand of CS) revealed a positive and statistically significant effect only with studies using specific pharmaceutical-grade products (nine studies) [15–22]; using data from these nine studies, the total number of patients treated with CS was 1009, and 1032 patients received a placebo. Eight of these studies were conducted in patients with knee OA and one included patients with hand OA [23]. Out of these eight studies, five also reported the effect of pCS on function [15, 16, 19–22].

Bourgeois et al. [15] conducted a three-arm, 13-week, placebo-controlled study where a single daily dose of 1200 mg pCS, given as an oral gel, was compared to 400 mg capsules t.i.d. in 127 patients with Kellgren–Lawrence (KL) score grade of I–III. Spontaneous pain, assessed by visual analogue scale (VAS) was significantly reduced compared to placebo (*p* < 0.05) with an SMD of − 0.90 (95% CI − 1.35; − 0.45). No significant differences were observed between the two active groups for pain reduction. In this study, function was appraised by the Lequesne Index (LI), a composite index which integrates pain and function [24]. A significant improvement in the LI was observed for the pCS groups (*p* < 0.01) but not in the placebo arm. The SMD for function effect versus placebo was − 0.84 (95% CI − 1.28; − 0.39).

Bucsi and Poor [16] followed 85 patients over 26 weeks. KL grades were I–III. Pain was assessed by VAS. Two capsules (400 mg each) were administered daily throughout the study. Pain was significantly reduced compared to baseline (*p* < 0.01) and versus placebo (*p* < 0.01) with an SMD vs placebo of − 0.92 (95% CI − 1.37; − 0.47). LI was assessed for both knees and a statistical difference versus placebo and versus baseline was observed in the group receiving pCS (*p* < 0.01 for both). SMD for LI reduction vs placebo was − 0.46 (95% CI − 0.89; − 0.03).

The primary endpoint of the study run by Kahan et al. [17] was the radiographic progression of knee OA, with symptomatic changes being assessed as a secondary outcome. 622 patients with KL grade I–III were enrolled and followed for 104 weeks. The intake of 800 mg of pCS, once daily, resulted in a significantly better improvement in pain [VAS and the pain subscale of the Western Ontario and McMaster Universities Osteoarthritis Index (WOMAC)] [25] than in the placebo group (*p* < 0.01 for the interaction between time and treatment effect by analysis of variance on ranks). Analyses conducted every 3 months of the study showed that for the decrease in pain (VAS), the difference between pCS and placebo was only significant between baseline and week 39 (*p* < 0.001). The SMD for pain reduction (VAS) at the end of the study was − 0.03 (95% CI − 0.19; 0.13).

300 patients with KL I–III score were enrolled for 104 weeks in the Michel et al. [18] study to determine whether pCS is effective in inhibiting cartilage loss in knee OA. The effect of a single daily 800 mg tablet of pCS on pain was assessed as a secondary end-point by measuring the WOMAC pain subscale, every 13 weeks. The intent-to-treat (ITT) analysis did not show any significant improvement of pain in the pCS group compared to placebo, with a SMD of − 0.19 (95% CI − 0.42; 0.04). The authors explained this result by the relatively low mean pain score at baseline, since the patients selection criteria did not include any minimum level of pain, which left little room for symptoms improvement.

The ChONdroitin versus CElecoxib versus Placebo Trial (CONCEPT) [19] was a three-arm study comparing in 604 patients (KL I–III) the effect of one tablet (800 mg) daily of pCS to an active comparator (celecoxib 200 mg/day) and to a placebo. Both active compounds showed, after 26 weeks, a significantly greater reduction in pain (VAS) compared with the placebo group (*p* = 0.001 for pCS and *p* = 0.009 for celecoxib), with no significant difference between the two active groups (*p* = 0.446). SMD for pain reduction was − 0.21 (95% CI − 0.41; − 0.02) for pCS. LI improved more in the two active groups compared to placebo after 13 and 26 weeks (significance at 26 weeks: *p* = 0.023 for pCS and *p* = 0.015 for celecoxib) while no difference was observed, at the end of the study, between pCS and celecoxib (*p* = 0.89). In the pCS group, the SMD for function improvement was − 0.16 (95% CI − 0.36; 0.03) at the end of the study.

In Uebelhart et al. [20] early pilot study, a small sample of 42 subjects (KL scores I–III) were treated for 52 weeks with two daily sachets, each containing 400 mg of pCS, which was compared to a placebo. Spontaneous pain (VAS) was significantly reduced at the end of the study, in the pCS group compared both to baseline and to placebo (both *p* < 0.01), with an SMD of − 1.06 (95% CI − 1.71; − 0.41). The same authors also reported (20) the effects of an intermittent administration of pCS (800 mg daily as one sachet) or placebo, for two periods of 13 weeks during 52 weeks. The study was conducted in 120 patients with symptomatic knee OA (KL scores I–III). Analyses of variance for multiple comparisons showed a significant difference between both treatment groups for pain (VAS) reduction after 9 and 12 months (*p* < 0.05). At the end of the study, SMD was reported as − 0.42 (95% CI − 0.79; − 0.04) for pain reduction. LI, which was the primary outcome of the study, decreased in both groups compared to baseline but the reduction was reported to be significantly greater in the pCS group than in the placebo group, after 39 weeks (*p* < 0.05) and after 52 weeks (*p* < 0.01). At this time point (52 weeks), the SMD for LI reduction with pCS vs placebo was − 0.32 (95% CI − 0.69; 0.08).

The equivalence of a single daily dose (1200 mg oral gel) compared to a three-time a day dose (400 mg capsules) of pCS was tested [22] over 13 weeks in 353 patients (KL not specified) with symptomatic knee OA. Both pCS groups were more effective than placebo in decreasing pain (VAS) (*p* = 0.02) with no difference between the two pCS regimens. Pain reduction at week 13 amounted an SMD of − 0.31 (95% CI − 0.57; − 0.06). A significant difference between the two treated groups and the placebo was observed for the LI after 8 weeks (*p* = 0.003) and 13 weeks (*p* = 0.0001) whereas the two groups receiving pCS improved similarly.

When including all these studies considered as presenting a low risk of bias and only using pCS, in our meta-analysis [13], we reached an overall significant effect for pain reduction (ES − 0.25; 95% CI − 0.34; − 0.16) and for improvement in function (ES − 0.33; 95% CI − 0.47; − 0.20).

### Safety of CS in OA

As reported by previous meta-analyses [26, 27], our recent meta-analysis on safety of various SYSADOAs has provided strong evidence that CS has a good safety profile [14]. In this new meta-analysis, only data from full safety reports were used for all the studies included in the analyses. This safety meta-analysis also distinguished between the studies using or not other anti-OA medications as rescue or concomitant medication. Both, in studies allowing concomitant anti-OA medications and in studies not permitting such concomitant medications, there were less patients reporting AEs in CS group compared with placebo; however, the odds ratio (OR) for total AEs (any AE) between CS and placebo was statistically significant only with studies not allowing concomitant anti-OA medications (OR = 0.70; 95% CI: 0.51, 0.98). There were no more AEs with CS compared with placebo in specific system organ classes investigated, including the gastrointestinal, vascular, cardiac, nervous system, skin and subcutaneous tissue system, musculoskeletal and connective tissue, and the renal and urinary system. Ultimately, this meta-analysis found no statistically significant increase in severe or serious AEs with CS, compared with placebo [14].

## Discussion

Chondroitin sulfate is a sulfated glycosaminoglycan composed of chains of alternating d-glucuronic acid and *N*-acetyl-d-galactosamine [9]. CS is available as pharmaceutical-grade (pCS) and nutraceutical-grade products, the latter exhibiting striking variations in preparation, composition and/or purity [13, 15–18]. Purity and/or production/purification of the CS compounds could orient the OA disease process either towards an inhibition or in the direction of a stimulation of the catabolic pathways inside the cartilage and may positively or negatively impact on collagen type II synthesis [28]. These differences may explain why pCS was shown to improve pain and function in several studies considered as being at low risk of bias, whereas other investigators using low-grade CS failed to do so. Brand, together with the risk of bias and the study size was recently shown to be the major determinant of the inconsistency observed in the results of studies assessing the symptomatic benefits of CS in knee OA [13]. Discrepancies between guideline documents on the role of pCS or pCGS for the management of knee OA may be also attributed to the fact that some of the guidance documents take into account this difference between pharmaceutical-grade and nutraceutical-grade CS or GS [5], while other guidelines consider only these products as a class with no attempt to separate the effects by grade or brand [3]. Eventually, in the Kahan et al. study [17] the WOMAC scores were translated into health utility index (HUI) allowing for the calculation of incremental cost-effectiveness ratio (ICER) of pCS compared to placebo [29]. Using the price bracket of pCS in Europe, ICER assessment always resulted in a cost below 30,000 € per quality-adjusted life years (QALY) suggesting that pCS treatment could be considered as cost-effective in patients with knee OA, up to a period of 24 months [29]. Using data from full safety reports, CS was also shown to have a good safety profile [14].

## Conclusion

In studies categorized as presenting a low risk of bias, pharmaceutical-grade CS was repeatedly and consistently shown to have a beneficial effect on pain and function in patients suffering from knee OA. The safety profile of pCS was shown, in recent meta-analysis using data from full safety reports, to be excellent. Furthermore, the use of pCS in the treatment of knee OA was found to be cost-effective up to a period of 24 months. Therefore, the results of these well-conducted studies support the recommendation to consider pCS as a first-background for the symptomatic treatment of mild-to-moderate (KL II–III) knee OA. However, no evidence exists that these results may be extrapolated to nutraceutical-grade preparations of CS [30].
